# Informal carers' support needs, facilitators and barriers in the transitional care of older adults: A qualitative study

**DOI:** 10.1111/hex.13596

**Published:** 2022-09-07

**Authors:** Jacqueline Allen, Michelle Lobchuk, Patricia M. Livingston, Natasha Layton, Alison M. Hutchinson

**Affiliations:** ^1^ School of Nursing and Midwifery Monash University Clayton Victoria Australia; ^2^ College of Nursing, Rady Faculty of Health Sciences University of Manitoba Winnipeg Manitoba Canada; ^3^ School of Nursing and Midwifery, Centre for Quality and Patient Safety Research, Institute for Health Transformation Deakin University Geelong Victoria Australia; ^4^ Rehabilitation, Ageing and Independent Living Research Centre, Peninsula Campus Monash University Frankston Victoria Australia

**Keywords:** informal carer, older adult, transitional care, user experience

## Abstract

**Introduction:**

Inclusion of informal carers in transitional care is challenging because of fast throughput and service fragmentation. This study aimed to understand informal carers' needs during the care transitions of older adults from inpatient care to the community.

**Methods:**

A qualitative exploratory design was used with mixed‐methods data collection. Seventeen semi‐structured telephone interviews were conducted with family carers; one focus group was conducted by videoconference with two family carers and three community‐based advocacy and aged care providers; and eight semi‐structured telephone interviews were undertaken with healthcare practitioners from rehabilitation services. Data were thematically analysed.

**Findings:**

All carers described the main social challenge that they needed to address in transitional care as ‘Needing to sustain family’. Carers reported their social needs across five solutions: ‘Partnering with carers’, ‘Advocating for discharge’, ‘Accessing streamlined multidisciplinary care’, ‘Knowing how to care’ and ‘Accessing follow‐up care in the community’. Focus group participants endorsed the findings from the carer interviews and added the theme ‘Putting responsibility back onto carers’. All healthcare practitioners described the main social challenge that they needed to address as ‘Needing to engage carers’. They reported their social solutions in three themes: ‘Communicating with carers’, ‘Planning with carers’ and ‘Educating carers’.

**Discussion:**

Findings highlight the importance of reconstructing the meaning of transitional care and relevant outcomes to be inclusive of carers' experiences and their focus on sustaining family. Transitional care that includes carers should commence at the time of hospital admission of the older adult.

**Conclusions:**

Future sustainable and high‐quality health services for older adults will require transitional care that includes carers and older adults and efficient use of inpatient and community care resources. Healthcare professionals will require education and skills in the provision of transitional care that includes carers. To meet carers' support needs, models of transitional care inclusive of carers and older adults should be developed, implemented and evaluated.

**Public Contribution:**

This study was conducted with the guidance of a Carer Advisory Group comprising informal carers with experience of care transitions of older adults they support and community‐based organizations providing care and advocacy support to informal carers.

## 1 INTRODUCTION

Informal carer support during older adults' transitions from hospital to the community is crucial to ensure safe care. Yet, inclusion of informal carers as part of older adult transitions is challenging because of the fast throughput and service fragmentation.^1^ Informal carers (carers) are those who provide unpaid support to older adults and others in the community and typically comprise family and friends.^2^ Transitional care is defined as the continuous and coordinated care for patients and carers across different health programmes and settings including discharge from hospital to home.^3^ When there is suboptimal involvement of carers in the transitions of older adults, this results in adverse outcomes including increased carer stress, unmet needs at home, unnecessary readmission to hospital and unwanted permanent placement in residential care.^4^, ^5^, ^6^ Few evidence‐based carer‐centred resources are available in transitional care of older adults because limited research has been conducted.^5^, ^7^ This study aimed to address this gap by describing carer roles and needs during the care transition of older adults to the community.

### 1.1 Transitional care for older adults and carers' support needs

Currently, transitional care research emphasizes outcomes for health services such as early readmission rates and length of stay.^8^, ^9^ In these studies,^8^, ^9^ researchers identified that compared with usual care, formal transitional care interventions including discharge assessment, planning, care coordination, medication reconciliation and self‐management reduce length of stay and readmission rates. Two well‐researched models used in the United States of America, the Care Transitions Intervention^3^ and the Transitional Care Model,^10^ have been influential in orienting health services towards the importance of self‐management and advanced practice nursing support. In Australia and in the United Kingdom, transitional care for older adults is characterized by multidisciplinary teams aiming to promote rehabilitation and care integration between inpatient and community providers.^11^, ^12^ Researchers have explored the outcomes of transitional care integrated with multidisciplinary and aged care teams and found reduced readmission rates and reduced functional decline in older adults.^4^

Despite these improvements in knowledge, these outcomes have not consistently translated into practice and the optimal role and involvement of carers during older adults' care transitions remain under‐researched. Consequently, there is a limited evidence base to guide health services in care and support for carers.^7^

Limited research has been conducted with carers. A systematic review of transitional care education interventions for carers of older adults who have experienced stroke found that education was effective in reducing carer burden and anxiety.^13^ In another systematic review, home‐ and outpatient‐based medication reviews in the follow‐up period after hospitalization improved carers' medication knowledge and self‐efficacy.^14^ Other studies have identified that carers need support with problem solving and decision‐making during the follow‐up discharge period,^15^, ^16^, ^17^, ^18^, ^19^ support to access and navigate community‐based programmes^20^, ^21^, ^22^ and support to clarify expectations of services.^23^ Some researchers have identified that carers can be ignored by healthcare practitioners during transitional care of older adults.^2^

Research to describe carer‐centred transitional care that includes carers in transitional care decision‐making would improve understanding of carer support needs. Future research is required to provide guidance that supports the growth of carer‐focussed health service systems and resources during the transitions of older adults from hospital to home.

## 2 RESEARCH AIM AND OBJECTIVES

This study aimed to understand carers' roles and needs during the care transition of older adults from inpatient care to the community. There were two research objectives:

1. To describe carers' roles and support needs.
2. To understand enablers and barriers to the inclusion of carers in transitional care from carers' and healthcare practitioners' perspectives.

## 3 METHODS

We used a qualitative exploratory design with mixed‐methods data collection. Data collection involved (1) 17 semi‐structured telephone interviews with carers; (2) one focus group conducted online using videoconference software with two carers and with three community‐based advocacy and aged care providers; and (3) eight semi‐structured telephone interviews with healthcare practitioners.

### 3.1 Conceptual framework

Transitional care of older adults is shaped through social interactions between older adults, carers and healthcare practitioners within social contexts of healthcare. We therefore adopted a social constructivist understanding of user experience. In this conceptual framework, user experience was viewed as a social process that is generated by people through their interactions with each other and with their social environment.^24^, ^25^ In this study, ‘users’ include carers supporting older adults receiving services and healthcare practitioners providing services. The main meaning unit for qualitative analysis was user experience.

We interpret inclusion of carers in transitional care as any user experience where carers took part in transitional care planning and decision‐making with healthcare practitioners in inpatient or community settings. Drawing on Spencer et al.,^26^ enabling and constraining factors were any experiences facilitating carers' involvement in transitional care or hindering their involvement.

### 3.2 Setting

This study was undertaken in the Australian community. Carer participants for the semi‐structured telephone interviews were recruited through community‐based carer advocacy and support services in the Australian states of Victoria, Western Australia, South Australia, Queensland and New South Wales. Carer participants for the focus group were recruited through the community‐based carer advocacy and support service in Victoria. Community‐based providers and representatives for the focus group were recruited from one community‐based carer advocacy group in Victoria and two culturally specific community organizations providing aged care for Greek and Italian communities. Healthcare practitioners were recruited from one large urban public healthcare network in Victoria and two national professional organizations representing nurses and social workers.

### 3.3 Ethics approval

Approval was obtained from the Monash University Human Research Ethics Committee for the semi‐structured interviews with carers and for the focus group. The Monash Health Human Research Ethics Committee provided ethics approval for the interviews with healthcare practitioners. An explanation of the study was guided by an Explanatory Statement as relevant to the semi‐structured interview or focus group. Participation in the study was voluntary. All participants provided written consent. All data were deidentified and all interviews were audio‐recorded for transcribing.

### 3.4 Semi‐structured interviews

#### 3.4.1 Carers

Carers were aged 18 years or older and were supporting an older adult aged at least 65 years and living in the community with multiple chronic health difficulties. Carers were required to have recent experience, within the last 2 years of a discharge from hospital to home following admission for a physical health problem of the older adult who they supported. Purposive sampling was used with maximum variation for ethnicity and geographic location including metropolitan and rural areas.

Initially, carers who fulfilled the selection criteria were recruited with the support of one participating carer advocacy and support organization in the Australian state of Victoria. Carers were invited to participate in a semi‐structured telephone interview and to contact the first author for additional information via an invitation provided in newsletters, social media and the websites of the participating organization. To optimize the saturation of data, we sought 25 carers. However, because of the COVID‐19 pandemic, and subsequent delays in recruiting carers, we expanded the recruitment base to carer advocacy and support organizations nationally. We also expanded our recruitment period. Carer participants were interviewed between March 2020 and October 2021. Across this period, we recruited 27 participants, with 10 declining to take part in an interview due to lack of time. This resulted in a total of 17 interviews with carers.

#### 3.4.2 Healthcare practitioners

Healthcare practitioners aged over 18 years of age who were providing transitional care to older adults in inpatient rehabilitation settings were eligible to participate in the study. Purposive sampling was used to select healthcare practitioners from nursing, allied health and medicine. Managers at the participating Victorian public health service in rehabilitation care were asked to provide recruitment information about the study to practitioners who fulfilled the inclusion criteria. Interested participants were invited to contact the first author for an explanation of the study. We originally sought 10 healthcare practitioner participants to triangulate healthcare practitioner interview data with that of carer participants. Due to the COVID‐19 pandemic and delays to research activities in the participating health service, we expanded our invitation to take part in the study in the newsletters, social media and websites of one national professional organization for nurses and of one national professional organization for social workers. We selected professional organizations for nurses and for social workers because nurses and social workers are frequently engaged in transitional care.^4^ In recruitment information, nurses and social workers were asked to contact the first author for an explanation of the study. We recruited nine healthcare practitioners, with one declining further participation due to lack of time. Eight healthcare practitioners were interviewed from August 2020 to November 2021.

#### 3.4.3 Data collection tools

Demographic questionnaires collected information about (1) the carer and the older adult being supported by the carer and (2) about healthcare practitioner participants (see Supporting Information: File SA). We developed the semi‐structured interview guides from an earlier scoping review of the literature (see Supporting Information: File SB).

#### 3.4.4 Procedure

Carers and healthcare practitioners who contacted the first author were sent the relevant Explanatory Statement and Consent Form for their consideration. With the carer's or healthcare practitioner's permission, the first author contacted them by telephone to explain participation in the study. Participants were asked to return the signed Consent Form to the first author either by email or by regular mail. Upon receipt of the signed Consent Form, the first author contacted the participant to make an appointment for an interview. All interviews were conducted by the first author by telephone. The first author is educated at post‐graduate level and is skilled in conducting semi‐structured interviews with research participants. Additionally, the first author is a female registered nurse, with skills in community nursing practice and experience in interviewing carers of older adult adults living with chronic disease and healthcare practitioners. The first author documented field notes following each interview to assist with data analysis.

### 3.5 Focus group

Because of recruitment challenges due to COVID‐19, we did not meet our recruitment plan to interview 25 carers. We therefore checked for saturation of categories by conducting one focus group with carers and with community‐based advocacy and aged care providers. In this focus group, we presented themes and subthemes from the semi‐structured interviews with carers to the focus group participants and invited additional feedback.

#### 3.5.1 Participants

The inclusion criteria for carers were the same as those followed for the interviews. Carers were recruited with the support of a carer advocacy and support organization in Victoria through an invitation provided in their social media. Interested carers were asked to contact the first author. The first author explained participation in the focus group and requested written consent. Additionally, we purposively sought participants from one community‐based carer advocacy organization and two culturally specific community‐based organizations providing aged care for Greek and Italian communities. We selected organizations supporting Greek and Italian communities as these are the most frequent older adult populations among migrant communities in Australia.^27^ Managers at each organization were sent an invitation explaining the study with a request for one member of their staff to participate. Interested participants contacted the first author, who explained the study to them and requested their written consent. In total, five participants took part in the focus group including two carers and three participants from community‐based organizations and aged care providers.

#### 3.5.2 Interview guide

In the focus group, we presented a summary of the themes from the findings in the semi‐structured interviews with carers. Following recommendations by Bate and Robert,^25^ we used the questions ‘Do the findings make sense? How/how not?’ to guide the discussion and collect any additional information.

#### 3.5.3 Procedure

There were public health restrictions preventing social gatherings because of the COVID‐19 pandemic. Therefore, the focus group was conducted and recorded online using video conferencing software. The first author facilitated the focus group with the support of a research assistant. The research assistant observed group dynamics occurring virtually and documented field notes to assist with data analysis.

### 3.6 Data analysis

Demographic information from data captured during semi‐structured interviews with carers and healthcare practitioners was tabulated. All interview recordings were transcribed for analysis. All qualitative interview data were thematically analysed by the first author using the inductive data analysis technique of thematic analysis. Thematic analysis was guided by the study aim and supported by the framework analysis method.^28^ Carer interviews, the focus group interview and healthcare practitioner interviews were analysed as three distinct groups. Codes were then compared and contrasted to develop categories and themes. Each interview transcript was coded independently by two coauthors. Raw data files (interview transcripts) were cross coded between the first author and one other coauthor as part of dependability and confirmability checks to derive a credible formulation of the data. The trustworthiness of the study methods was supported by the triangulation of each data set.^24^

## 4 FINDINGS

**Figure 1:**
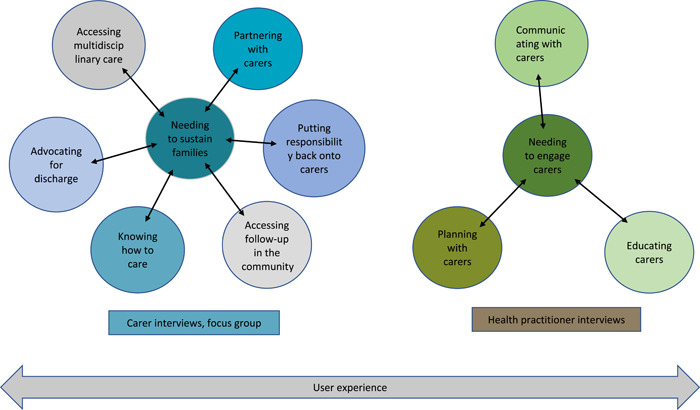
Summary of themes carer interviews, focus group and health practitioner interviews.

A total of 30 carers, healthcare practitioners and representatives from community and peak body organizations took part. We present the findings from the semi‐structured interviews with carers, the focus group with carers and representatives from community and peak body organizations and semi‐structured interviews with healthcare practitioners. A summary of findings from each data collection method is shown in Figure 1.

### 4.1 Semi‐structured interviews with carers

All 17 carer participants were family carers and most (14) were female. Carers were aged between 44 and 80 years. Carers' education ranged from Year 9 secondary school to a master's degree at university. Most carer participants (12) resided in urban areas and five resided in rural and regional Australia. All carers spoke English and five spoke a first language other than English. Demographic information of carers is presented in Table 1.

**Table 1:** Carer characteristics

Participant	Sex	Age	Residential state/territory	Urban/rural/regional	Language spoken at home	Highest level of education	Occupation	Relationship to older adult	Carer's health
P01	F	50	Victoria	Urban	Hungarian	Year 11 College (TAFE) Certificate	Full‐time carer Retired laboratory technician	Daughter	Nil reported difficulties
P02	F	80	Victoria	Urban	English	Year 12	Retired court officer	Spouse	Nil reported difficulties
P03	F	74	Victoria	Urban	English	Bachelor's degree	Retired Information technology consultant	Spouse	Diabetes Chronic heart condition
P04	F	59	Victoria	Rural	English	Master's degree	Manager government department	Spouse	Hypertension Psoriasis Arthritis
P05	M	76	South Australia	Urban	English	Year 11 College (TAFE) Certificate	Retired truck driver	Spouse	Prostate cancer
P06	F	44	Victoria	Urban	Greek	Bachelor's degree	Community youth worker	Granddaughter	Anxiety Depression
P07	F	61	New South Wales	Urban	English	Bachelor's degree	Disability support consultant	Daughter	Arthritis Depression Fibromyalgia
P08	F	71	New South Wales	Regional	English	Year 9 College (TAFE) Certificate	Retired office worker	Spouse	Chronic back pain Mobility difficulties
P09	F	49	Victoria	Urban	Russian	Bachelor's degree	Administration and management	Daughter	Arthritis
P010	F	65	Western Australia	Rural	English	Year 9	Small business owner Clothing designer	Spouse	Hypothyroidism
P011	F	51	Victoria	Urban	English	Year 10	Full‐time carer	Daughter	Back problems Arthritis Fibromyalgia
P012	F	51	Queensland	Rural	English	College (TAFE) Certificate	Community support worker	Daughter	Fibromyalgia Chronic pain Hip injury (through caring)
P013	F	53	Western Australia	Urban	English	Graduate certificate	Leadership role higher education	Daughter	Nil reported difficulties
P014	M	65–75	Victoria	Urban	English	Master's degree	Teacher	Son	Nil reported difficulties
P015	F	49	Western Australia	Urban	English	Year 12	Full‐time carer Part‐time waiter	Daughter	Bipolar disorder Posttraumatic stress disorder Rheumatoid arthritis Chronic pain
P016	M	68	Western Australia	Urban	English, Maori	Year 9 College (TAFE) Certificate	Carpenter	Spouse	Nil reported difficulties
P017	F	44	Western Australia	Rural	Telugu, Hindi	Bachelor's degree	Full‐time carer	Spouse	Alcohol misuse

The 17 carers supported an older adult who had experienced at least one care transition from an inpatient setting to their own home within 2 years of the interview. Older adults receiving carer support ranged in age from 71 to 92 years and most (11) were female. Demographic information about older adults receiving carer support is presented in Table 2.

**Table 2:** Characteristics of persons being supported at home

Carer participant	Sex	Age	Residential state/territory	Urban/rural/regional	Language spoken at home	Receives aged pension	Occupation	Living arrangements	Older adult's health
P01	F	86	Victoria	Urban	Hungarian	Age pension	Retired laboratory technician	Lives alone	Chronic obstructive pulmonary disease Heart failure Anxiety Depression Glaucoma
P02	M	83	Victoria	Urban	English	Part age pension	Retired electrical engineer	Lives with carer	Heart disease Renal disease Motor neurone disease Type 2 diabetes
P03	M	76	Victoria	Urban	English	Nil	Retired surveyor	Lives with carer	Renal disease Chronic pain Degenerative problems in the spine
P04	F	83	Victoria	Rural	English	Nil	Retired farmer	Lived with carer while in transition from hospital	Hypertension Hypothyroidism Polymyalgia Chronic urinary tract infections Venous leg ulcers Heart failure Parkinson's disease
P05	F	77	South Australia	Urban	English	Age pension	Retired unskilled worker	Lives with carer	Bowel cancer Metastatic cancer
P06	F	92	Victoria	Urban	Greek	Age pension	Retired unskilled worker	Lives with carer's parents	Stroke Recurrent falls Deafness
P07	F	84	New South Wales	Urban	English	Age pension	Retired medical secretary	Lives with carer	Type 1 diabetes Peripheral neuropathy Macular degeneration Parkinson's disease Arthritis Depression Breast cancer Incontinence
P08	M	73	New South Wales	Regional	English	Age pension	Retired truck driver, painter	Lives with carer	Lung cancer Recent hip surgery
P09	F	71	Victoria	Urban	Russian	Disability support pension	Home duties	Lives with spouse	Type 2 diabetes Hypertension Neuropathy Arthritis Chronic pain Ischaemic heart disease
P010	M	76	Western Australia	Rural	English	Age pension	Retired unskilled worker	Lives with carer	Dementia Arthritis Chronic obstructive pulmonary disease
P011	F	83	Victoria	Urban	English	Age pension	Retired manager caravan park	Lives with carer	Arthritis Mobility difficulties Deafness Asthma
P012	F	78	Queensland	Rural	English	Age pension	Renovation and house sales	Lives alone	Falls Wheelchair bound Degenerative disk disease Type 2 diabetes Neuropathy Dementia Incontinent
P013	M	77	Western Australia	Urban	English	Age pension	Unskilled worker	Lives alone	Cancer of the bladder and prostate Major pelvic surgery and complications of surgery (fistula) Urostomy bag Heart failure
P014	F	91	Victoria	Urban	English	Age pension	Retired small business owner	Lives alone	Rheumatoid arthritis Impaired vision Atrial fibrillation
P015	F	78	Western Australia	Urban	English	Part age pension	Retired office assistant	Lives alone	Neck pain Degeneration of cervical vertebrae Recurrent surgery to neck and significant loss of function (walking, use of arms and hands
P016	F	75	Western Australia	Urban	English	Age pension	Retired laboratory technician	Lives with carer	Stroke Left hemiplegia Fully dependent on carer for personal care
P017	M	76	Western Australia	Rural	English	Age pension	Retired driver	Lives with carer	Stroke Fully dependent on carer for personal care

All carers described the main social challenge that they needed to address in transitional care of the older adult as ‘Needing to sustain family’. Carers reported five themes describing facilitators: ‘Partnering with carers’, ‘Accessing streamlined multidisciplinary care’, ‘Advocating for discharge’, ‘Knowing how to care’ and ‘Accessing follow‐up care in the community’. Participant carers further explained barriers within each of the five themes. Quotes illustrating each theme are presented in Table 3.

**Table 3:** Carer interviews: Main themes, subthemes and quotes

Quotes
*Needing to sustain family*
Subtheme 1: Transitioning to a family carer role
‘With my mother growing up we did not have a wonderful relationship because her attention was on my brother who was always ill. My brother was more important. I didn't have that close relationship with my mother. I have built the relationship over the last 5, 7 years. Which I cherish’. Participant 9
‘We've been together 34 years and we've lived together 24 hours a day, seven days a week, and loved it all. So the idea of him going somewhere else and me going off on my own is absolutely ridiculous … I don't want to leave him. The most important thing for me, right now, is being together and embracing what time we have’. Participant 10
Subtheme 2: Valuing caring
‘When we married, it was in sickness and health. There's a moral and ethical obligation for me to fulfill that now. It's the way we were brought up, ethical and moral values. He's my husband. Provided for my children, has been a very good husband I feel glad I can do this for him now’. Participant 2
Subtheme 3: Needing that break
‘It does get you a bit isolated. It does depress you a bit. I go to a mental health carers group. Used to go shopping which was exercise and a break – can't do that anymore because of COVID 19’. Participant 8
*Partnering with carers*
Subtheme 1: Involving carers
‘Yes, they did [involve me in discharge planning], and the doctors would often call me, if I wasn't there, and discuss things with me, with my mum’. Participant 14
‘It's like “don't really want to deal with this anymore [care of the older adult]. I find it rather dismissive. … I don't feel that they talk a lot to the family. I don't feel that they [acute care system] involve you”’. Participant 1
Subtheme 2: Deciding the discharge
‘He, in my opinion was quite ‐ still quite unwell and should not have been discharged. … I don't believe we were ‐ we should ‐ I think we should have been consulted about, is he in a ready state and is the family in a ready state to accept him home’. Participant 13
‘Once they decide to discharge, it's all done very efficiently and very fast. A little bit too fast at times’. Participant 3
*Accessing streamlined multidisciplinary care*
Subtheme 1: Communicating with multidisciplinary teams
‘Frustrating, because yeah, so mum was admitted a number of times here. … she'd had massive ulcers on her leg, had had the fall, 24 hours out to it, no idea what was going on. Yet the hospital persisted in having discussions with mum about her care and what was going to be happening and not relaying that information through me’. Participant 4
‘They did not know my circumstances. I had shoulder surgery in July a shoulder repair. So I was dealing with that. It would have been good if they had checked’. Participant 7
Subtheme 2: Planning coordinated discharge care
‘There's five different departments he might get treatment from – I don't know how much they talk to one another. … As soon as he comes home and starts eating, blood levels will all change again? This guy [pharmacist] had not considered that. So a bit disappointing. He came home and I hadn't been able to speak to anybody. The next day he's vomiting again. You go into emergency, and they only look at one thing. They go to a ward, only look at their area of responsibility’. Participant 3
Subtheme 3: Accessing culturally competent care
‘No matter how much I ask that there needs to be either an interpreter or there needs to be me as a family member to understand what we need to do after the discharge, it never happens, ever. It just never happens’. Participant 9
*Advocating for discharge*
Subtheme 1: Negotiating care
‘The [health service] does take complaints seriously … they would check that my facts were correct … then someone will ring me and say, well, let's make an appointment’. Participant 9
‘So, I am very much fighting [as a consumer representative] for carers to be given the respect of a proper clinical handover and not just be given this person to take home’. Participant 12
‘Instead of answering my question, she said, if you want to talk to us doctors, here, you need to be here at eight when we do our rounds’. Participant 17
Subtheme 2: Being confident to advocate
‘My mum knew to be assertive and say I can't have her back. She is not mobile yet. … I gave them a few words to say’. Participant 6
‘Frankly, it's only because I'm more educated and have been through the health system a few times now with my mum and myself, I kind of understand the language… I feel a little bit more confident in order to question and be a bit more of an advocate and assertive around what it is we need’. Participant 13
*Knowing how to care*
Subtheme 1: Learning discharge care
‘I got in touch with the doctor. Who said do this, this and this. I devised a method for myself. I measured his ankle and calves to see what size, if it started to creep up, I'd increase the dose [of diuretics]’. Participant 2
‘They all say [inpatient providers] to her [mother] how lucky she is to have me because I can work it out on my own [carer titrates insulin for her mother]’. Participant 12
Subtheme 2: Accessing discharge information
‘There is the pamphlets. …. I try to read what he's been through, what he's had and his levels on the treatment’. Participant 8
‘There was nothing, not once was I ever really told fully what [wife's name] conditions were, like described in layman's terms. … I asked for a copy of the discharge notes,… all written in medical jargon which I wouldn't understand’. Participant 5
‘No information about care needs … and I was horrified that taking somebody home with this ghastly – what was for me, as a lay person, a hideous wound that I was quite terrified of. So I said to the discharge nurse, can you tell me what I'm supposed to do? He went, oh, it's all in there [wound care package]….a lot of what he said that should have been in there wasn't in there.’ Participant 10
‘He [father] was sent home with this catheter with no written instruction about what to do, … it was all a little bit rushed. … the catheter stopped working at some point over the next few days and my dad didn't realise that that was an issue … I said right we're going to the hospital. It turned out that basically he was on the verge of sepsis and because he hadn't had any instructions about what to do or what sepsis looks like … he ended up in hospital’. Participant 13
*Accessing follow‐up care in the community*
Subtheme 1: Referring to community care
‘No, nothing, nothing. Not a thing. [Health service] Didn't ask if I needed any help, nothing. … Mum has got an aged care package [name of service provider] and we've got a new case manager … she contacted me two weeks after Mum had had the surgery; do you need anything, do you need any extra help or anything? No, not now, I'm good’. Participant 11
‘We received the level 4 package … I sourced a provider and … I've been running that now … I can't fail them on the discharge because they've got a protocol they've got to work, and they do it every time… It worked for me’. Participant 16
Subtheme 2: Communicating with community care
‘We have a fantastic relationship with our aged care provider, so absolutely. I messaged Mum's case manager straight up when she went into hospital. He maintained contact with me throughout the week and when she came out, he checked whether anything needed to be changed. Thankfully, this time no, but yes, they were very active’. Participant 9
‘I felt very dismissed in my opinions [with community allied health practitioner]….I didn't feel that what I was saying, because it wasn't what was on the list, had any value. … You should listen to that person because they're doing the caring’. Participant 10
Subtheme 3: Navigating community care
‘They're [carers organisation] a really valuable source of information … we've got another aged care assessment, to try and get a package… the Carer's Organisation have said that they would be able to try and assist to get mum's package through a bit quicker because I've got the caring role in two directions. They've got funding, they've got advice, they're supportive’. Participant 1
‘I never felt that there was a case management process that actually made it happen’. Participant 4
‘They sent an assessor out to speak to [wife] and I about what we needed, what would happen, did we need this, did we need that, do we need something else … they told us what they could get us’. Participant 5

#### 4.1.1 Theme 1: Needing to sustain family

All carers commented that sustaining family relationships was important to them during the transitional care of the older adult because caring roles are part of what constitutes a family. Carers further reported that the role of a carer can itself challenge ‘normal’ family relationships. All carers explained that discharge and care transition episodes of the older adult are stressful and challenge their family relationships. They noted that they required support to resume ‘normal’ relationships through improved transitional care. They described the theme of Needing to sustain family in three subthemes: (1) Transitioning to a family carer role; (2) Valuing caring; and (3) Needing that break. All carers explained that sustaining family relationships with the older adult required them to transition from being a family member to being a family carer. The seven spousal carers reported that despite the older adult's dependence, they remain in a spousal relationship and continuing companionship at home was important to them. Another carer commented that he was honouring his promise to his wife by caring for her at home. All other 10 participants reported that they wanted the best transitional care and health for the older adult so that they could resume quality family relationships. One carer explained quality family relationships in further detail. She supported her father across multiple readmissions to hospital with sepsis. She stated that she wanted community support and stable health for her father so that she could return to a ‘normal’ father–daughter relationship. Fifteen carers commented that caring caused them to worry about the older adult and that on occasion they needed a break from caring. Seven carers explained that transitional care caused them additional worry and at times distress during COVID‐19 lockdowns because they were prevented from visiting the older adult and because outpatients and community follow‐up services were cancelled.

#### 4.1.2 Theme 2: Partnering with carers

Family carers considered Partnering with carers in two subthemes: (1) Involving carers and (2) Deciding the discharge. Five carers reflected that healthcare practitioners involved them as partners in transitional care. Nine carers, on the other hand, reported that healthcare practitioners did not involve them in transitional care. Another three carers emphasized their mixed experiences across multiple care transitions of the older adult over the previous 2 years. According to one carer, healthcare practitioners in acute care did not involve the carer; however, healthcare practitioners from subacute care involved the carer. Another carer commented that the healthcare practitioners in acute care involved her in discharge planning; however, in other admissions over the past 2 years, healthcare practitioners in acute care did not involve her in discharge planning for her mother. One other carer explained that healthcare practitioners at the urban rehabilitation unit where her husband was cared for following his stroke did not involve her or communicate with her in transitional care planning. She explained that her husband was subsequently transferred to a rural hospital close to her home at short notice; however, the rural healthcare practitioners involved her in her husband's discharge planning.

All carers explained that they had limited roles in decisions about when the older adult would be discharged. One carer commented that the hospital staff always ensured that the older adult was well before they discharged him home to her care. According to two other carers, with support from nursing staff and from social workers, they were able to negotiate with the health service for the older adult to remain in hospital to ensure that all possible support was available at home. Another two carers explained that the older adult they supported was discharged quickly, and they had little time to prepare the home. Two other carers commented that the older adult was discharged prematurely, resulting in early readmission to hospital.

#### 4.1.3 Theme 3: Accessing streamlined multidisciplinary care

Carer participants described the theme Accessing streamlined multidisciplinary care in three subthemes: (1) Communicating with multidisciplinary teams; (2) Planning coordinated discharge care; and (3) Accessing culturally competent care. All carers reflected that effective communication and coordination were essential to streamlined multidisciplinary care. Three carers reported that they were satisfied with discharge communication. In contrast, six carers commented that they were not satisfied with discharge communication because it occurred only between the healthcare practitioner and the older adult, resulting in errors in discharge medications, care coordination and the availability of equipment at home. Four other carers explained that they had limited communication with healthcare practitioners and another three carers reported that they were not provided with a discharge handover. Two carers stated that they were not asked about their availability to support the older adult and one of these carers was unable to provide care because of a shoulder injury.

Four carers reported that the discharge was well coordinated, and another four carers reported that care was not coordinated. Three carers further explained their experiences of limited coordinated medical care when the older adult required specialist medical care from multiple teams. Another carer reported poor coordination between the allied health team, the acute ward and the aged care respite facility.

Two carers reflected their perception that access to culturally competent care was part of streamlined multidisciplinary discharge. One carer explained that her grandmother was unable to access Greek interpreters in both public and private hospitals during several care transitions. According to this carer, during her grandmother's recent hospital discharge, she had access to Greek interpreters, indicating culturally competent care. However, another carer reported that although her mother accessed a Russian interpreter about medication changes at discharge, an interpreter was not available for other areas of discharge planning. Because the family was not contacted about the discharge, the older adult did not understand her follow‐up care requirements.

#### 4.1.4 Theme 4: Advocating for discharge

Carers reflected on the theme Advocating for discharge in two subthemes: (1) Negotiating care and (2) Being confident to advocate. Sixteen carer participants reported that they attempted to negotiate transitional care with healthcare practitioners. Four carers commented that the older adult did not speak up for themselves and therefore the carer needed to negotiate on their behalf. Another carer noted that she ensured that one family member was always present with her grandmother to ensure that discharge negotiation took place in a culturally competent manner. Two other carers commented that the older adult could negotiate care with carer support. One of these carers reported that she would reiterate her mother's statements to support her mother to negotiate with healthcare practitioners for what she required.

Eight carers explained that they used questioning and discussion to negotiate transitional care with healthcare practitioners. Four carers reported that they discussed modifications to discharge treatments and three carers negotiated for additional allied healthcare following discharge. Another two carers reported that they negotiated social worker support for the older adult in transitional care to improve their communication with the care team. One carer commented that she insisted on a clinical handover of her mother's follow‐up care so that she could provide quality care at home. Two other carers reported that they questioned medications with healthcare practitioners to ensure accurate discharge treatment. Another three carers noted that despite their attempts to negotiate discharge care, some healthcare practitioners did not listen to them. Two of these carers reported that when healthcare practitioners ignored them, they used the formal complaints system at the health organization as a strategy to negotiate transitional care. As one carer explained, following her complaint, her mother received follow‐up outpatient care and was diagnosed with an acute myocardial infarction that was not diagnosed during her mother's hospital admission.

Eight carers explained that they needed confidence to effectively negotiate and advocate for the older adult and that they learned to do this from previous hospital discharge experiences. According to one carer, she coached her parents to be assertive and confident with healthcare practitioners in the discharge of her grandmother.

#### 4.1.5 Theme 5: Knowing how to care

Participant carers described Knowing how to care in two subthemes: (1) Learning discharge care and (2) Accessing discharge information. Six carers reported that they learned how to navigate discharge care following advice from inpatient healthcare practitioners, community‐based allied healthcare practitioners and from their carer support organization. One carer, in caring for his wife following her stroke, conducted searches of the internet to supplement what he learned from healthcare practitioners. However, six carers reported that they received inadequate education about discharge care, resulting in carers not knowing how to care. Three carers further explained that because they did not know how to care, two older adults experienced complications with wound healing and one older adult was readmitted to hospital with an infection.

Eight carers explained how they accessed written discharge information. Five carers reported that inpatient healthcare practitioners provided them with written information about services and who to contact in the event of problems following discharge. Another two carers reported that they asked their general practitioner (GP) to send them a copy of the discharge summary, although the GP did not always have a discharge summary. Another carer noted that she supported her father in accessing information about his self‐care at home after discharge by ‘dumbing down’ the medical jargon on his discharge medication instructions. This carer suggested that these instructions should be more pictorial for older adults to improve understanding. Eight other carers reported their experiences of barriers to accessing discharge information about the older adult including inconsistent discharge information and being ignored by healthcare practitioners.

#### 4.1.6 Theme 6: Accessing follow‐up care in the community

Participants described Accessing follow‐up care in the community in three subthemes: (1) Referring to community care; (2) Communicating with community care; and (3) Navigating community care. All carers reported mixed experiences in accessing follow‐up care at home because of the fragmentation of health and aged care services. Nine carers explained that they experienced effective communication with community services at follow‐up. Twelve carers reported that referrals to community care were not necessary because they already had services at home. According to these carers, their existing community care support was responsive and timely, although one carer commented that the community service was inadequate for her father's complex nursing care needs. Another carer explained that her mother was referred to Hospital in the Home and to the local council; however, this resulted in confusion about which service was responsible for care at home and errors resulted. One other carer reported that she was not offered any support at home from either the inpatient service or the community aged care case manager.

Seven carers discussed the value of social workers and allied health to undertake assessments at home and avoid gaps in follow‐up care. Two carers noted that they were unable to access physiotherapy reviews in the home and that inpatient practitioners advised them to arrange for private physiotherapists. Another carer reported that the inpatient practitioners required him to find a high‐care respite bed for his wife at short notice to free up the bed.

Four carers commented on the valuable support from an allied health professional about navigation of community supports. Eight carers reported that community‐based organizations assisted them in navigating services and another three carers reported that the Aged Care Assessment Services supported them in navigating community services. One carer noted that the Aged Care Assessment Service was not able to advise her about navigating care for her husband who lived with dementia. Another two carers reported that the community aged care case managers did not contact them and one of these carers consequently decided to take over her mother's case management.

### 4.2 Focus group

Following presentation of the themes from the interviews with carers, all five focus group participants endorsed the findings. Focus group participants, including two family carers and three community‐based advocacy and aged care providers, added the theme ‘Putting responsibility back onto carers’. In this theme, all participants described their experience that the health and aged care services discharged older adults too early and provided inadequate community support for carers. All participants reflected that the health and aged care systems use informal unpaid carers to support older adults to save money for health and aged care services.

### 4.3 Healthcare practitioners

Eight healthcare practitioners took part in an interview. Seven participants were female. Participants' ages ranged between 30 and 59 years. All participants were employed in public health services located in eastern and south‐eastern Australia. Table 4 presents the demographic information.

**Table 4:** Healthcare practitioner characteristics

Participant	Age	Gender	Discipline	Language spoken at home	Highest qualification	Setting	Current role	Length of time in occupation
01	30–39	Male	Nursing	English	Master's degree	Victoria, urban, inpatient rehabilitation	Associate Nurse Unit, Manager and Clinical Support Nurse	8 years
02	30–39	Female	Medicine	Tamil	Postgraduate diploma	Victoria, urban, inpatient rehabilitation	Consultant geriatrician	9 years
03	40–49	Female	Social work	English	Postgraduate diploma	Victoria, urban, inpatient rehabilitation	Ward social worker	15 years
04	30–39	Female	Pharmacy	Mandarin Chinese	Master's degree	Victoria, urban, inpatient rehabilitation	Clinical pharmacist	16 years
05	40–49	Female	Social work	English	Bachelor's degree	Australian Capital Territory, urban, rehabilitation (inpatient and community)	Transitional care programme Social worker	16 years
06	20–29	Female	Occupational therapy	English	Bachelor's degree	Victoria, urban, rehabilitation (inpatient‐based, home visits)	Rehabilitation programme Occupational therapist	4 years
07	50–59	Female	Nursing	Sinhalese	Postgraduate certificate	Victoria, urban, inpatient palliative care	Educator palliative care	11 years
08	50–59	Female	Social work	Filipino	Bachelor's degree	Queensland, rural, rehabilitation (inpatient and community)	Intensive rehabilitation programme Senior social worker	6 years

All healthcare practitioners described the main social challenge that they needed to address in care transitions in the theme ‘Needing to engage carers’. They reported their social solutions to this problem in three themes: ‘Communicating with carers’, ‘Planning with carers’ and ‘Educating carers’. Quotes illustrating each theme are presented in Table 5.

**Table 5:** Healthcare practitioner interviews: Main themes and illustrative quotes

Quotes
*Needing to engage carers*
‘It's really crucial to involve carers and the whole system really, the whole system that's supporting that person who has been an inpatient’. Health practitioner 5: Social Worker
‘In this setting [rehabilitation care]… a range of different cultures and backgrounds, without family support, often it can be challenging to work with patients and maximise their function to a level where it is safe for them to return home. I think having that family support really aids in that process’. Health practitioner 6: Occupational Therapist
*Communicating with carers*
‘I don't … get any sort of idea of what they're like in the community without the collateral history. I can't do any sort of assessment without contact with the person who they live with or care for. In geriatrics, you do a lot of digging to find the truth of the matter’. Health practitioner 2: Geriatrician
‘My understanding their emotional state as well, I spend a lot of time with the carers on discharge planning and I listen to what they are thinking about planning’. Health practitioner 7: Registered Nurse
*Planning with carers*
‘So sometimes the discharge engagement process is me having those really, really hard conversations in family meetings. Once all of my colleagues have presented the evidence and I try and be really structured in family meetings to enable them [family carers] to feel like we've got control of the situation. Because it is a very out‐of‐control feeling to be placed in a position where you are wanting something that maybe you can't facilitate’. Health practitioner 3: Social Worker
‘I am also supporting the medical team and the Allied Health because they don't really know what to do [because of the conflict in the family]’ Health practitioner 8: Social Worker
‘I think that should be really clear to an informal carer what they're signing up for and that we want their preferences and the patient's preferences, but also what that responsibility is so that they can actually tell what they're taking on’. Health practitioner 1: Registered Nurse
*Educating carers*
‘We then get the permission from the patients whether they're happy for us to contact their family. … Usually, they are quite happy for us to do that. It's just that I like to have an extra ear for them. I usually will go through the medication information with the patient as well as the family or carers. So that everybody's on the same page’. Health practitioner 4: Pharmacist
‘This family member was just furious that we couldn't complete the session of haemodialysis, and just an explanation to say that their blood pressure couldn't allow it ‐ they're, like, well then what are you doing? I'm, like, we actually cannot do this. It was really just a lack of understanding about basic physiology and about the limitations of that person's body and multisystem dysfunction. No matter how many times you repeated that conversation and tried to come at it from different angles, sometimes the health literacy also translated into lower levels of acceptance’. Health practitioner 2: Geriatrician
‘We get carers to come in and actually learn how to do things, either to build insight into the fact that well actually this person was caring for you so you can't care for them … we'll let you try so that you can realise that that's not possible’. Health practitioner 1: Registered Nurse

#### 4.3.1 Theme 1: Needing to engage carers

All participants explained that they need to engage carers in care transitions because they form the older adult's support system in the community. All participants further explained that the demand for beds put them under pressure to discharge older adults quickly and this was a barrier to engaging with carers. According to seven participants, other barriers included conflict within the family and limited available time. Two participants reported that carers do not always respond to telephone calls, and this was another barrier to engaging them in transitional care. Three participants further explained that engaging carers from culturally diverse and low socioeconomic backgrounds is challenging because these families require additional support and resources. Five participants reported that despite their increased use of telephone and video conferencing, COVID‐19 lockdowns were a significant barrier to engaging carers who were not able to visit the inpatient setting.

#### 4.3.2 Theme 2: Communicating with carers

All participants explained that communication was essential to building relationships with carers. According to three participants, they engaged carers in discussion about the prognosis of the older adult to enable carers to plan for postdischarge care. Three participants commented that social workers are particularly skilled in communicating with carers because of their ability to accommodate different perspectives of family members. Two participants reported that nurses required improved communication skills to better engage carers. Seven participants commented that they learned communication skills as part of their initial education in their discipline. All participants explained that they learned skills in engaging with carers through role modelling from senior clinicians.

#### 4.3.3 Theme 3: Planning with carers

All healthcare practitioner participants reported that multidisciplinary teams should include carers in transitional care planning to optimally prepare the home for the older adult's return. Seven participants explained that planning with carers best occurred informally at the bedside, or when required, in formal family meetings. According to one participant, family meetings enable discussion with multiple family carers, which was useful for complex discharge planning of older adults with high‐care needs.

Seven participants explained the need to plan risk management in discharge for older adults with the carer/s and family to avoid unnecessary readmission to hospital. Another participant explained that carers needed to understand the responsibility of agreeing to provide follow‐up care at home. According to one participant, despite risk identification and planning with carers, older adults with high‐care needs were often readmitted early to hospital. Two healthcare practitioners commented that lack of community‐based services and lack of access to professional nurses in the community were barriers to carers' support during the follow‐up period. According to another participant, follow‐up support for carers was challenging in rural communities because staff were not available.

#### 4.3.4 Theme 4: Educating carers

All participants reported that engaging carers in practical education about the older adults' personal care and medications was important in a successful discharge. Three participants explained that practical education of carers should involve learning how to navigate emergency and community‐based services. Two participants noted that effective education should include carers taking care of themselves. Another three participants reported that low health literacy was a barrier to educating carers. These participants explained that low health literacy could contribute to conflict between carers and healthcare practitioners. Another two participants noted that unnecessary readmissions to hospital could occur when carers did not understand education provided by healthcare practitioners.

## 5 DISCUSSION

The findings identify the social features and processes central to the inclusion of carers in transitional care, including facilitators and barriers, from a social constructivist perspective of user experience. According to carer interview participants, sustaining family was the main social problem that they encountered and being excluded from decision‐making in transitional care was a substantial barrier. Being a partner in transitional care, accessing multidisciplinary care, advocating for discharge, knowing how to undertake follow‐up care and accessing community‐based follow‐up care were the social solutions and facilitating processes that they used. These findings indicate that keeping the family together, rather than the hospital experience, was carer participants' most important focus. Focus group participants endorsed the findings from the carer interviews and added the theme ‘Putting responsibility back onto carers’, where they perceived that government restricted inpatient‐ and community‐based healthcare funding for older adults, leaving carers with greater accountability for older adults' health and care in the community. Although healthcare practitioners described ‘Needing to engage carers’ in older adults' care transitions and facilitating factors of communication, planning and education, they also emphasized that demand for fast bed throughput was a significant barrier.

All interview themes addressed essential communication skills that healthcare practitioners require to include carers in older adults' care transitions. Communication skills included those pertaining to therapeutic support, culturally safe and competent care, exchange of information and quality multidisciplinary care and education and carer health literacy. Other studies^15^, ^18^, ^20^ have also identified the vital role of healthcare practitioners' communication skills in the care transitions of older adults. However, the findings from the current study indicated a power imbalance between family carers and healthcare practitioners, which was a barrier to communication and to including carers in the transitional care of older adults. The demand for inpatient beds resulted in early discharge and limited time and resources to engage carers in transitional care planning. There are significant consequences for carers, older adults and for healthcare services of increased demand for inpatient beds and early discharge including heightened carer burden, unwanted placement in residential aged care and unnecessary admission to hospital.^5^ Interview findings with carers were characterized by carer stress and included examples where uncoordinated care and limited community‐based care contributed to the older adult's placement into permanent care. Inadequate carer education at discharge played a part in an early readmission of the older adult.

In previous studies emphasizing health services' efficiencies in transitional care research, the focus was on the management of inpatient bed demand.^8^ Previous research in transitional care has largely defined effective transitional care in institutional terms, for example, where transitional care is defined as discharge planning and preparation.^8^ The institutional focus in transitional care research is particularly notable in health services' outcomes and measures of efficiency such as readmission rates and length of stay.^8^, ^9^ Findings from the current study highlight the importance of reconstructing the meaning of transitional care and outcomes to prioritize carers' experiences and their focus on sustaining family.

Previous research has identified that carers require support with problem solving and decision‐making in the postdischarge period^15^, ^16^, ^17^, ^18^, ^19^ alongside improved access to community support and community service navigation.^20^, ^21^, ^22^ Findings from the current study expand on this knowledge. Carers emphasized facilitating factors of being partners in transitional care, accessing multidisciplinary care, advocating for discharge and knowing how to undertake follow‐up care. Each of these social processes occurred while the older adult was an inpatient. In tandem with the current focus on Day 1 of admission on discharge planning, the inclusion of carers in transitional care should commence with the older adult's hospital admission.

The theme of accessing community‐based follow‐up care included carers' reports of challenges with service navigation, care coordination and service integration. This indicates unmet needs for some carers during the follow‐up period. Australia, like many other western countries, has undergone a policy and funding shift to consumer‐directed models of community‐aged care and subsequent increases in responsibility for carers.^29^ There are many benefits of these models such as greater choice and control over decision‐making for older adults.^30^ However, echoing previous research,^29^, ^31^ the current findings identified that consumer‐directed care models have changed the support needs of carers. Carers increasingly require assistance with service navigation and coordination during the follow‐up period in care transitions.

Findings from this Australian study and from previous international studies^15^, ^18^, ^20^, ^32^, ^33^, ^34^ indicate that transitional care that includes carers of older adults in decision‐making is ad hoc. Findings from these studies^15^, ^18^, ^20^, ^32^, ^33^, ^34^ suggest that in western countries, transitional care requires redesign to consistently meet the support needs of carers and older adults to remain living in the community in accordance with their wishes. Some health practitioners' inconsistent and ad hoc approach to including carers and older adults in decision‐making during care transitions suggests that patient throughput and bed management may be their primary considerations. This is also mirrored in the transitional care research where indicators of health services' efficiencies are emphasized.^8^ However, quality transitional care is a multidimensional practice construct where multiple priorities require attention. Transitional care systems and practices that support the inclusion of carers and older adults in decision‐making are imperative alongside efficiencies for health services.

### 5.1 FURTHER RESEARCH

Findings from the current study indicate that the high demand for hospital beds, variable quality of systems of transitional care and differing skills of healthcare practitioners are barriers that need to be overcome. Findings also indicate the strengths of carers and of healthcare practitioners. Research is required to develop and test resources supportive of carers from diverse groups with varying levels of health literacy. Importantly, systems of transitional care incorporating carers and older adults require development and evaluation.

### 5.2 LIMITATIONS

There are several limitations to the current study. All carer participants were family carers; therefore, the perceptions of nonfamily carers of older adults were not captured. We included carers supporting older adults with multimorbidity. Carer support needs may vary for older adults with specific health conditions, and we may not have captured this information. We are unable to comment on issues in the data influenced by local conditions and health systems because we sampled carers across Australia. Therefore, the current findings describe carers' experiences of transitional care across a range of Australian health services. We included eight healthcare practitioners and we cannot claim data saturation. All healthcare practitioners were employed in rehabilitation, and the perceptions of healthcare practitioners in other settings were not ascertained. However, healthcare practitioner interviews were undertaken as part of data triangulation. We argue that these interviews provided additional insight about the rehabilitation practice environment including enabling and constraining factors that carers may encounter during care transitions. This information may be of use to others in similar contexts of care elsewhere.

## 6 CONCLUSIONS

Future health services policy regarding older adults will depend upon transitional care that includes carers as part of routine practice in decision‐making. Healthcare practitioners will require quality communication skills to deliver transitional care with carers and older adults. This will require increased focus on the preregistration education of healthcare practitioners. Carer involvement in care transitions will be essential to sustainable high‐quality care for older adults alongside efficient use of inpatient resources including the management of inpatient beds. To address carers' support needs, models of transitional care should be developed, implemented and evaluated using participatory methods with carers as experience experts and as educators of policy makers, health services planners and healthcare practitioners.

## AUTHOR CONTRIBUTIONS

Jacqueline Allen, Michelle Lobchuk, Patricia M. Livingston and Alison M. Hutchinson contributed to the study design and conceptualization. Jacqueline Allen led the data analysis and formulation of the data, and all coauthors contributed to the coding and data analysis. Jacqueline Allen led the writing of the manuscript, with contributions from all coauthors. All authors reviewed and approved the final manuscript.

## ACKNOWLEDGEMENTS

The authors gratefully acknowledge all carer, healthcare practitioner and community organization participants in this study. The authors acknowledge the support of Carers Victoria, CO‐AS‐IT, Pronia, Carers Western Australia, Carers New South Wales, Carers Queensland, Carers South Australia, Carers Tasmania, National Rural Health Alliance, Consumers Health Forum, Monash Health, Thorne Harbour Health, the Australian College of Nursing and the Australian Association of Social Workers. The authors acknowledge the assistance of Dr Marta Woolford. This study was proudly supported by the Australian Association of Gerontology research trust.

## CONFLICT OF INTEREST

The authors declare no conflict of interest.

## Supporting information

Supplementary information.

Supplementary information.

## Data Availability

Data sharing is not applicable.
