# Supplementary material for: Informal carers' support needs, facilitators and barriers in the transitional care of older adults: A qualitative study
Source: Health Expect. 2022 Sep 7;25(6):2876–92. doi: 10.1111/hex.13596 (PMC9700166; doi:10.1111/hex.13596)
Supplement: Supplementary file 1 — Supplementary information. [file HEX-25--s002.docx]

**Supplementary File A**

Data collection tool: Demographic information carers and older adults

| **Carer** | |
| --- | --- |
| Age |  |
| Gender |  |
| Place of birth |  |
| Speaks a language other than English at home |  |
| Education |  |
| Occupation |  |
| Receives government pension |  |
| Is an informal (unpaid) carer (family member, friend, neighbour) of an older adult living at home |  |
| Carer’s relationship to the older adult? |  |
| Carer living arrangements (with the older adult, with family/friends, alone) |  |
| Chronic health conditions |  |
| **Older adult being cared for / supported at home** | |
| Age |  |
| Gender |  |
| Place of birth |  |
| Speaks a language other than English at home |  |
| Occupation |  |
| Receives aged pension |  |
| Older adult’s living arrangements (with the carer, with family/friends, alone) |  |
| Chronic health conditions (older adult) |  |
| Medical / other care providers at home (for the older adult) |  |
| Older adult being supported by the carer has had at least one hospital admission in the previous two (2) years |  |

Data collection tool: Demographic information healthcare practitioners

| Date of birth  (Or note age range) | 20-29, 30-39, 40-49, 50-59, 60-69, 70+ |
| --- | --- |
| Gender |  |
| Place of birth |  |
| Speaks a language other than English at home |  |
| Qualifications |  |
| Occupation |  |
| Length of time in occupation |  |
| Current role |  |
| In what area of patient care do you work/what patient care unit/program? |  |
| Length of time in current role |  |
| What is the length of experience you have engaging with carers in discharge or transitional care? |  |
