# Supplementary material for: Informal carers' support needs, facilitators and barriers in the transitional care of older adults: A qualitative study
Source: Health Expect. 2022 Sep 7;25(6):2876–92. doi: 10.1111/hex.13596 (PMC9700166; doi:10.1111/hex.13596)
Supplement: Supplementary file 2 — Supplementary information. [file HEX-25--s001.docx]

**Supplementary File B**

Semi-structured interview questions: Carers

1. Can you tell me about the family member/friend that you support?

*Prompts:*

- 1. Tell me about their health and the care that they need to stay living in their own home in the community.
  2. (If relevant) What was the impact of your input as a carer in the home care plan?

1. Can you tell me about the support that you provide to your family member/friend?

*Alternative to Question 2:* Can you take me through an ordinary day?

*Prompt:* Can you tell me how much time you spend in caring?

1. What is the most important thing about caring for your family member/friend?

*Prompt:*

- 1. What do you value most about caring for your family member/friend?

1. Tell me about the most recent visit your family member/friend made to hospital (as an inpatient)?
2. Tell me about the discharge of your family member/friend from hospital back home?

*Prompts*

- 1. Were you included in the discharge planning process/by the healthcare team in the discharge planning process? If so, how were you included?
  2. Did you understand the care that your family member/friend needed after discharge?
  3. Did you receive all of the information that you needed to continue to provide support at home?
     1. What information did you receive (written, verbal, other)?
     2. What information was missed?
  4. Were you satisfied with the way that the hospital discharged your family member/friend? Why/Why not?
  5. What was your experience with the health care team in hospital? Re:
     1. Access to the team / contacting the team
     2. Being involved in discharge planning
     3. Consistent messages
  6. What services came to visit at home?
  7. Was your input as a carer actively sought out by the home care service? If so, how?
  8. What was important about the support you received at home after discharge?
  9. Were you satisfied with the way that the community providers supported you at home after the discharge of your family member/friend?
     1. How did home care meet your expectations? (Please expand on your answer)

1. What was done well in the discharge process?
2. What things were missing in the discharge process?
3. What was the most important thing about the discharge?

Semi-structured interview questions: Healthcare practitioners

Can you tell me about engaging informal carers (family, friends, neighbours) in discharge and transitional care of older people from hospital to home?

1. What is your experience of engaging with carers about discharge planning for older adults?
2. How do you engage with informal carers re hospital discharge?
3. How might engaging with informal carers be useful? Prompt: How might it be of value?
4. What skills and training do you have in engaging with informal carers re hospital discharge?
5. In your experience, can you describe what has been the best thing that has happened in engaging with informal carers during older adults’ discharge/care transition?
6. What is the worst thing that has happened in engaging with informal carers during older adults’ discharge/care transition?
7. What would help you to engage with informal carers during older adults’ hospital discharge/care transition? Prompts: What facilitates engaging with informal carers during older adults’ care transitions? What are the barriers to engaging with informal carers during older adults’ care transitions?
8. Lastly, is there something that I should have asked you about supporting informal carers re discharge and transition of older adults from hospital to home that I have not?
9. Is there anything else about your experience that you think is important for us to know?
